# Diet‐induced obesity alters skeletal muscle fiber types of male but not female mice

**DOI:** 10.1002/phy2.204

**Published:** 2014-01-28

**Authors:** Maxwell S. DeNies, Jordan Johnson, Amanda B. Maliphol, Michael Bruno, Annabelle Kim, Abbas Rizvi, Kevyn Rustici, Scott Medler

**Affiliations:** 1 Department of Biology, State University of New York at Fredonia, Fredonia, 14063, New York; 2 Department of Biological Sciences, University at Buffalo, Buffalo, 14260, New York

**Keywords:** Fiber types, obesity, skeletal muscle

## Abstract

Skeletal muscles are highly plastic tissues capable dramatic remodeling in response to use, disuse, disease, and other factors. Growing evidence suggests that adipose tissues exert significant effects on the basic fiber‐type composition of skeletal muscles. In the current study, we investigated the long‐term effects of a high‐fat diet and subsequent obesity on the muscle fiber types in C57 BLK/6J mice. Litters of mice were randomly assigned to either a high‐fat diet or a control group at the time of weaning, and were maintained on this diet for approximately 1 year. Single fibers were harvested from the soleus and plantaris muscles, and fiber types were determined using SDS‐PAGE. The high‐fat diet mice were significantly heavier than the control mice (39.17 ± 2.7 g vs. 56.87 ± 3.4 g; *P* < 0.0003), but muscle masses were not different. In male mice, the high‐fat diet was associated with a significantly lower proportion of slow, type I fibers in the soleus muscle (40.4 ± 3.5% vs. 29.33 ± 2.6%; *P* < 0.0165). Moreover, the proportion of type I fibers in the soleus of male mice was inversely proportional to the relative fatness of the male mice (*P* < 0.003; *r*^2^ = 0.65), but no association was observed in female mice. In male mice, the decline in type I fibers was correlated with an increase in type I/IIA hybrid fibers, suggesting that the type I fibers were transformed primarily into these hybrids. The reported trends indicate that type I fibers are most susceptible to the effects of obesity, and that these fiber‐type changes can be sex specific.

## Abstract (precis)

Mice were fed a high‐fat diet and subsequently became obese. Obese male mice exhibited a significant decline in slow type I fibers in the soleus muscles, compared with controls. The loss of type I fibers was directly proportional to measures of body fat, suggesting a “dose”‐dependent effect on muscle phenotype.

## Introduction

Skeletal muscles are recognized as being some of the most highly plastic tissues, capable of dramatic remodeling in response to use, disuse, disease, and other factors. Whole muscles are heterogeneous in their composition, being built as a mosaic of different fiber types with distinct physiological properties. Muscles are commonly classified by the specific myosin heavy chain (MHC) isoforms they express. In mammals, at least 11 different MHC isoforms are encoded by separate genes (Schiaffino and Reggiani 2011). Of these, only four different MHCs are routinely expressed within the adult limb and axial muscles: I, IIA, IIX, and IIB (Schiaffino and Reggiani 2011). In humans, the gene encoding the IIB isoform is present, but is not expressed within the skeletal muscles to any extent (Schiaffino and Reggiani 2011). Although the specific fiber type composition of muscles is established genetically, transformations of fiber type in response to a variety of physiological parameters induce functional adaptations for a muscle's specific needs (Pette 2006; Schiaffino and Reggiani 2011). Precisely how much of a muscle's phenotype is predetermined by genetics and how much is a result of exercise or other factors remains uncertain (Schiaffino and Reggiani 2011). However, studies of human muscles have suggested that roughly 40% is genetically determined, but as much as 45% of fiber‐type proportions may be determined from the muscle's environment and patterns of usage (Simoneau and Bouchard 1995).

There is growing interest in the important linkages between skeletal muscles and adipose tissues (Petersen et al. 2007; Baldwin et al. 2011; Nielsen and Christensen 2011), particularly in the face of the impending worldwide diabetes epidemic (Zimmet et al. 2001). Most studies examining the effects of obesity and diabetes on skeletal muscles have come from researchers focused specifically on these diseases. Skeletal muscles are one of the major metabolic engines of the body, and studies have clearly shown that obesity and associated conditions like metabolic syndrome and diabetes cause an impairment of the oxidative capacity of the skeletal muscles (Petersen et al. 2007). In this context, it is essential to understand how skeletal muscle dysfunction contributes to these diseases, but it is also important to know how these conditions affect the muscles themselves. From the perspective of skeletal muscle plasticity, obesity provides an interesting model to consider how parameters like energy availability and adipose tissues can influence muscle structure and function. In the current study, we were interested in the basic biology of how diet and obesity might affect skeletal muscle organization and muscle fiber types. Our hypothesis was that diet‐induced obesity would cause a shift in muscle fiber type, with slower types being converted into faster ones.

Studies from human skeletal muscles have consistently shown that obesity is associated with a shift toward faster fiber types, with the proportions of slow fibers being inversely correlated with body fat levels (Lithell et al. 1981; Lillioja et al. 1987; Wade et al. 1990; Mårin et al. 1994; Hickey et al. 1995; Kriketos et al. 1996, 1997; Nyholm et al. 1997; Helge et al. 1999; Gaster et al. 2001; Tanner et al. 2002; Oberbach et al. 2006; Stuart et al. 2013). These patterns have led to the general hypothesis that increased adipose tissue levels drive fundamental changes in muscle fiber composition, which in turn lead to impaired metabolic function. Perhaps surprisingly, our basic knowledge of how skeletal muscles change with obesity in rodent models is limited, and many of the results of previous studies have been contradictory. Studies of diet‐induced obesity in mice have reported changes in the oxidative capacity of skeletal muscles, but with minimal or nonexistent changes in fiber type (Turner et al. 2007; de Wilde et al. 2008, 2009; Shortreed et al. 2009; Trajcevski et al. 2013). In contrast to human studies, obese leptin knockout (*ob/ob*) mice exhibit reduced muscle mass and a possible shift toward slower fiber types (Almond and Enser 1984; Stickland et al. 1994; Tankersly et al. 1998; Warmington et al. 2000; Kemp et al. 2009). The variability in these results likely stem from differences in study design, but significant differences may also exist between the human and rodent responses to obesity. Clearly, further studies of the effects of diet and obesity on mouse skeletal muscles are warranted. Although rodent muscles have some basic differences from human muscles, these experimental models provide a level of control not possible with human studies. Therefore, it is essential to develop a solid understanding of how dietary fats and obesity affect skeletal muscles in these animals.

**Figure 1.**
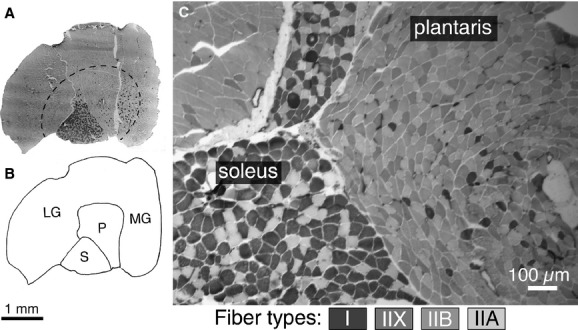
Anatomical location and fiber types present in mouse soleus and plantaris muscles. (A) Low magnification image and map (B) of the entire triceps surae complex: gastrocnemius (medial: MG and lateral: LG), plantaris (P), soleus (S). The great majority of fibers (>95%) of the fibers in the gastrocnemius outside the hatched line are type IIB in the mouse. (C) Higher magnification image of the soleus and plantaris to illustrate the basic fiber types present in these muscles. The soleus primarily comprised type I and IIA fibers, whereas the plantaris contains a mixture of fast fiber types. Staining intensity of different fiber types in the mouse is indicated in the key below, and is consistent with previous reports (Hamalainen and Pette 1993; Glaser et al. 2010). A and B are reproduced from Glaser et al. 2010.

In the current study, we randomly assigned C57 BLK/6J mice from three litters to either a high‐fat diet or to a control group. The high‐fat diet derived 60% of its calories from fats, whereas the control diet contained 18% of calories from fat. The mice were maintained on these diets for approximately 12 months before their muscles were collected for analysis. We then used single fiber SDS‐PAGE (sodium dodecyl sulfate polyacrylamide gel electrophoresis) to precisely determine the fiber types from the predominantly slow soleus, and the primarily fast plantaris muscles. In combination with the gastrocnemius, these deep shank flexors constitute the triceps surae complex. Collectively, the soleus and plantaris comprised all of the adult fiber types normally found in rodent limb muscles (Fig. 1).

Mice on the high‐fat diet became significantly heavier than the controls, but their skeletal muscles of the triceps surae complex were not different in mass. Compared to female mice, the proportions of slow type I fibers were fewer in the soleus of males. Males also exhibited significant differences in the proportions of type I fibers between control and obese mice. The relative proportions of type I fibers in the soleus were significantly smaller in obese compared to control males, and these proportions were inversely correlated with measures of body fat. These patterns are consistent with those observed in human muscles, and suggest a dose‐dependent reduction in type I fibers as a function of adipose tissue levels.

## Materials and Methods

### Ethical approval

C57 BLK/6J were maintained in the Laboratory Animal Facility at the University at Buffalo in accordance with an approved IACUC protocol. Mice were originally purchased from Jackson Laboratories, but were then bred to produce the young mice used in the current experiments. All mice were provided with food ad libitum and had continual access to water. Animals were euthanized by exposure to CO_2_ before tissues were collected for analyses.

### Animals and diet

Twenty‐three male and female mice from three litters were randomly assigned to the high‐fat diet group or the control group at the time of weaning (approximately 21 days old). The control diet delivered 18% of its calories in the form of fat (Teklad diet 2018), whereas the high‐fat diet provided 60% of its calories from fat, primarily in the form of saturated fats (Teklad diet 06414). Mice were fed ad libitum on these diets for approximately 1 year (363 ± 23 days). Mice were euthanized and the triceps surae muscle complex (consisting of the soleus, plantaris, and gastrocnemius) were dissected free, placed in a glycerination buffer (*composition described below*), and stored at −20°C.

### Animal and muscle masses

Animals were weighed to the nearest 0.1 g on a top loading balance. Individual muscles of the triceps surae complex were isolated from one another and weighed on an analytical balance to the nearest 0.1 mg. As a relative measure of animal adiposity, we standardized animal mass to the mass of the triceps surae complex. We termed this parameter animal “fatness,” and it corrects for inherent differences in mouse mass that are independent of the amounts of fat.

### Determination of MHC isoforms within single fibers

Individual muscle fibers were dissected from the isolated soleus and plantaris muscles. These muscles were selected for study because they possess a similar anatomical location within the triceps complex, and together they provide a good representation of all of the fiber types common in adult mouse limb muscles (Fig. 1). Fibers were not sampled from the gastrocnemius because the large majority of fibers in this muscle are uniformly type IIB (Fig. 1A and B) (Glaser et al. 2010). Isolated muscles were stored at −20°C in a glycerination buffer containing 50% glycerol, 2 mmol/L EGTA, 1 mmol/L MgCl_2_, 4 mmol/L ATP, 10 mmol/L imidazole, and 100 mmol/L KCl. Bundles of fibers were separated from whole muscles and placed in cold glycerination buffer, and individual fibers were separated from the bundle using fine forceps with the aid of a stereomicroscope and then placed in a microcentrifuge tube containing 30 μL of sample buffer. Sample buffer contained 8 mol/L urea, 2 mol/L thiourea, 50 mmol/L Tris base, 75 mmol/L dithiothreitol, 3% SDS and 0.004% bromophenol blue, pH 6.8 (Blough et al. 1996). Fiber samples were stored at −20°C until the time when they were prepared for SDS‐PAGE.

SDS‐PAGE resolving gels consisted of 9% acrylamide (200:1 acrylamide/methylene‐*bis*‐acrylamide), 12% glycerol, 0.675 mol/L Tris base (pH 8.8), and 0.1% SDS. Stacking gels consisted of 4% acrylamide (20:1 acrylamide/methylene‐*bis*‐acrylamide), 0.125 mol/L Tris base (pH 6.8), and 0.1% SDS. Running buffer contained 0.192 mol/L glycine, 25 mmol/L Tris base, 0.1% SDS, and 0.08% 2‐mercaptoethanol. Gels were run with a constant current of 20 mA for ~41 h at 8°C (Zhang et al. 2010). At the end of the run, gels were fixed with 50% MeOH containing 0.037% formaldehyde for at least 3 h at 4°C before staining. Gels were then quickly rinsed in deionized water for ~5 min before being stained with silver (Wray et al. 1981). Stained gels were soaked in 2% glycerol for 30 min and then air dried between cellophane.

### Histochemical staining

Muscle sections stained for myofibrillar ATPase are presented in the current study to demonstrate the anatomical organization of the muscles studied (Fig. 1. The entire triceps surae complex was rapidly frozen in isopentane cooled in liquid N_2_ and frozen sections (8–10 μm) were made using a Leica cryostat. Sections were mounted on glass slides and stained for ATPase according to the procedures of Dubowitz and Sewry (2007), as we have reported previously (Glaser et al. 2010; Zhang et al. 2010). These stained sections were not from the mice analyzed in the current experiment, but were from other ongoing studies of C57 BLK/6J mice in our laboratory. All of the statistical analyses in the current study were determined from single fiber SDS‐PAGE data.

### Statistical analyses

Body mass, animal “fatness,” and specific muscle masses were compared with *t*‐tests. Fiber‐type proportions within the soleus and plantaris were compared between high‐fat diet and control mice with *t*‐tests. Similar comparisons were also made between male and female mice. In addition, the relative proportions of fiber types were compared by factorial analysis of variance (ANOVA) where sex and diet were used as independent variables. Finally, regression analyses were used to determine correlations between fiber‐type proportions and continuous variables including body mass, relative “fatness,” and other fiber‐type proportions. Statistical significance was accepted at *P* < 0.05. Values are reported as mean ± SE.

## Results

### Body and muscle mass

**Figure 2.**
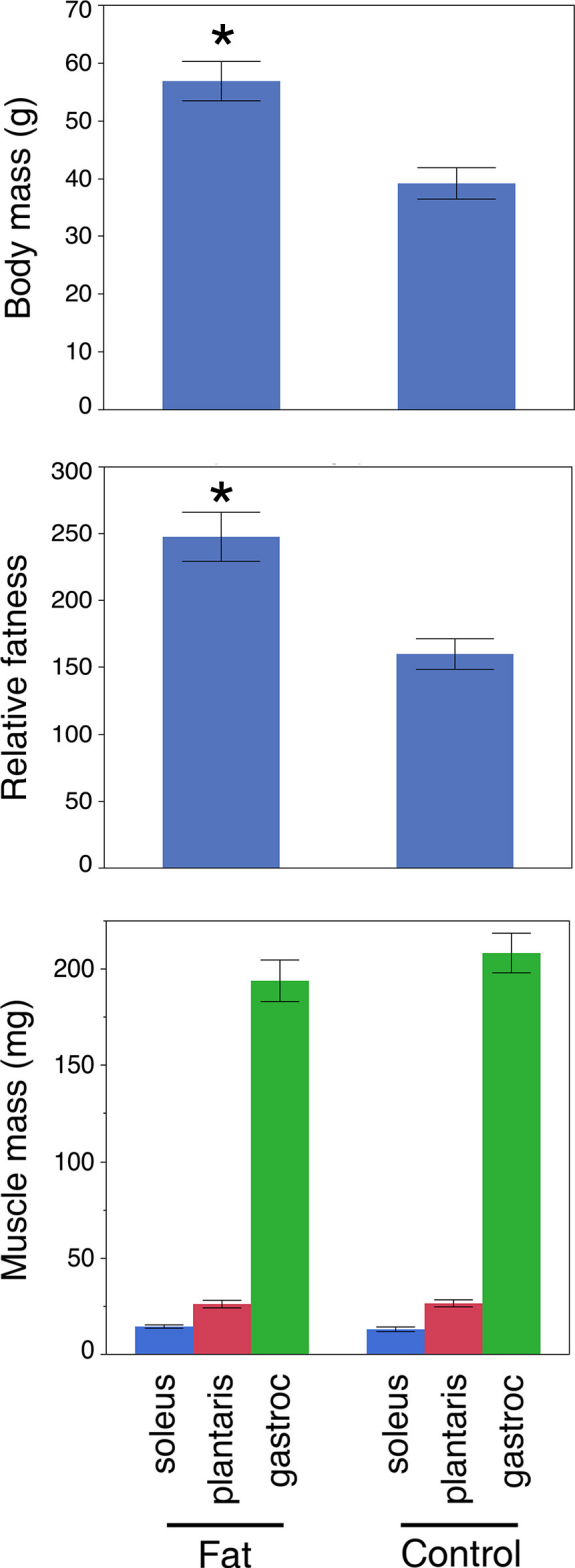
High‐fat diet induced an increase in mouse body mass but not muscle mass. (A) Mice fed a high‐fat diet (Fat) had a significantly higher body mass than controls (Control) (56.87 ± 3.4 g vs. 39.17 ± 2.7 g; *P* < 0.0003). (B) Relative fatness (body mass:triceps surae mass) was also significantly greater in the mice maintained on high fat. (C) Mass of the muscles comprising the triceps surae complex was not different between the high‐fat diet and controls.

Mice maintained on a high‐fat diet were significantly heavier than mice on the control diet (56.9 ± 3.4 g vs. 39.2 ± 2.7 g; *P* < 0.0003) (Fig. 2A). Relative “fatness” (body mass:triceps surae mass) was also greater in the high‐fat diet mice (247.8 ± 18.3 g/mg vs. 160.1 ± 11.4 g/mg; *P* < 0.0004) (Fig. 2B). No differences in any of the muscles of the triceps surae (soleus, *P* > 0.48; plantaris, *P* > 0.92; and gastrocnemius, *P* > 0.35) were found between treatment groups (Fig. 2C). The average muscle mass was greater in males than females for the combined gastrocnemius and plantaris (242.1 ± 11.1 mg vs. 213.4 ±10.9 mg; *P* > 0.0.04), but not for the soleus muscles (*P* > 0.50).

### Fiber‐type composition of soleus and plantaris muscles

**Figure 3.**
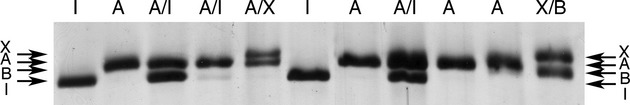
SDS‐PAGE used to identify single fiber types. Single fibers were dissected from whole muscles and loaded on SDS‐PAGE gels. The adult MHC isoforms migrated in the pattern IIX < IIA < IIB < I (migration direction from top → bottom). Individual fiber types from a soleus sample are identified above by their migration patterns.

**Figure 4.**
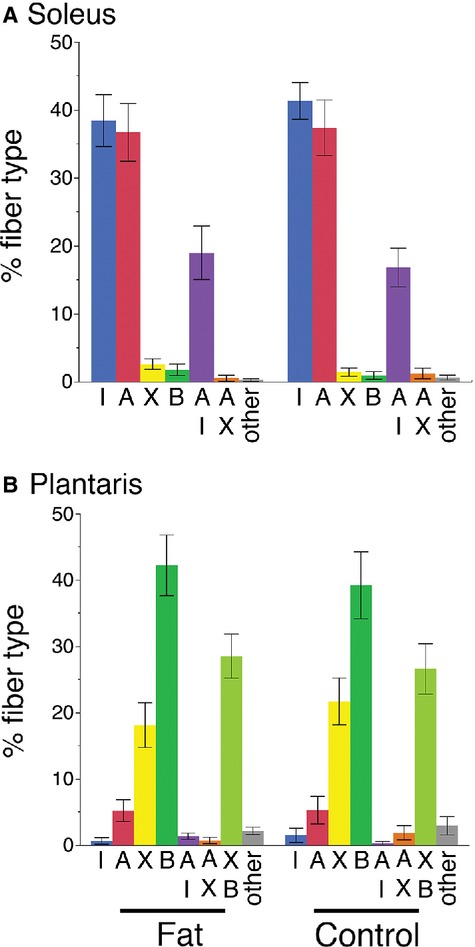
Skeletal muscle fiber types identified in soleus and plantaris muscles of high‐fat diet and control mice. (A) Soleus muscles have the highest proportions of types I, IIA, and I/IIA hybrids. Minor amounts of other fiber types are also present. When data from male and female mice are combined, no differences between high‐fat diet and control mice are evident. (B) Plantaris muscles primarily contain types IIB, IIX/IIB hybrids, and IIX fibers. No differences in fiber type between the high‐fat diet and control mice were detected within the plantaris.

Single fiber SDS‐PAGE provided clear resolution of MHC isoforms (Fig. 3). Under the specified running conditions, the four adult isoforms migrated in the order: I > IIB > IIA > IIX as we have reported previously (Zhang et al. 2010). We analyzed approximately 50 fibers from each muscle, for a total of 2577 fibers (1450 from soleus and 1127 from plantaris). The overall composition for the soleus muscle (*ordered from most to least abundant*) was as follows: I: 39.8 ± 2.4%; IIA: 37.1 ± 2.9%; I/IIA: 18.0 ± 2.5%; IIX: 2.2 ± 0.5%, IIB: 1.43 ± 0.53%; other hybrid fibers: 0.45 ± 0.2% (Fig. 4A). For the plantaris, the fiber‐type composition was skewed toward faster fibers, IIB: 40.8 ± 3.3%; IIX/IIB: 27.7 ± 2.5%; IIX: 19.9 ± 2.4%; IIA: 5.3 ± 1.3%; IIA/IIX: 1.3 ± 0.6%; I: 1.1 ± 0.6%; I/IIA: 0.9 ± 0.3%; other hybrid fibers: 2.6 ± 0.7% (Fig. 4B). These fiber‐type proportions are in general agreement with the proportions anticipated from histochemical staining of whole muscle sections (Fig. 1), and provide a comprehensive sample of the different fiber‐type combinations commonly found in mouse muscles.

### Effects of diet and sex on fiber‐type composition

**Figure 5.**
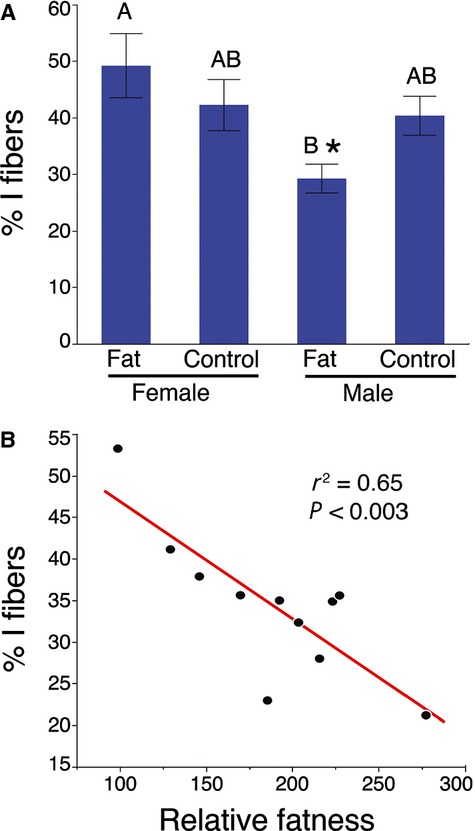
Fat male mice have reduced numbers of type I fibers. (A) Male mice maintained on a high‐fat diet had significantly fewer type I fibers than control male mice (*t‐test, *P* < 0.0165). Letters indicate the results of the Tukey post‐ANOVA test, where means with the same letter is not significantly different. (B) A significant correlation was observed between relative “fatness” and the proportion of type I fibers in male mice (*r*^2^ = 0.65; *P* < 0.003).

When analyzed as a group with both males and females included, no diet‐specific differences in fiber types were detected in either the soleus or plantaris muscles (Fig. 4). However, when the data were analyzed for both diet and sex effects, differences in the proportions of type I fibers in the soleus muscles became apparent. A factorial ANOVA detected significant differences in the abundance of type I fibers as a function of sex and in the interaction between sex and diet (Table 1). A Tukey pair‐wise post‐ANOVA test showed that the control diet female mice had significantly more type I fibers than the high‐fat diet male mice (Fig. 5A). Alternatively, a *t*‐test indicated that among male mice the high‐fat diet group contained significantly fewer type I fibers than the control group (Fat: 29.3 ± 2.6% vs. Control: 40.4 ± 3.5%; *P* < 0.0165) (Fig. 5A). In addition, the proportion of type I fibers was inversely proportional to the relative fatness of the male mice (%I = 60.88 − 0.14 × “fatness”; *r*^2^ = 0.65; *P* < 0.65; *P* < 0.003) (Fig. 5B).

**Table 1.** ANOVA of % type I fibers in soleus muscle.

Factor	df	Sum of squares	Mean square	*F* ratio	*P*‐value
Sex	1	621.40	621.40	7.15	0.016*
Diet	1	22.43	22.43	0.26	0.618
Sex × Diet	1	423.86	423.86	4.87	0.041*
Residual	17	1478.28	86.96		

Dependent variable: % type I fibers in the soleus; Sex: male or female; Diet: high‐fat diet or normal diet; Sex × Diet: interaction between sex and diet.

* Statistically significant: *P *<**0.05.

**Figure 6.**
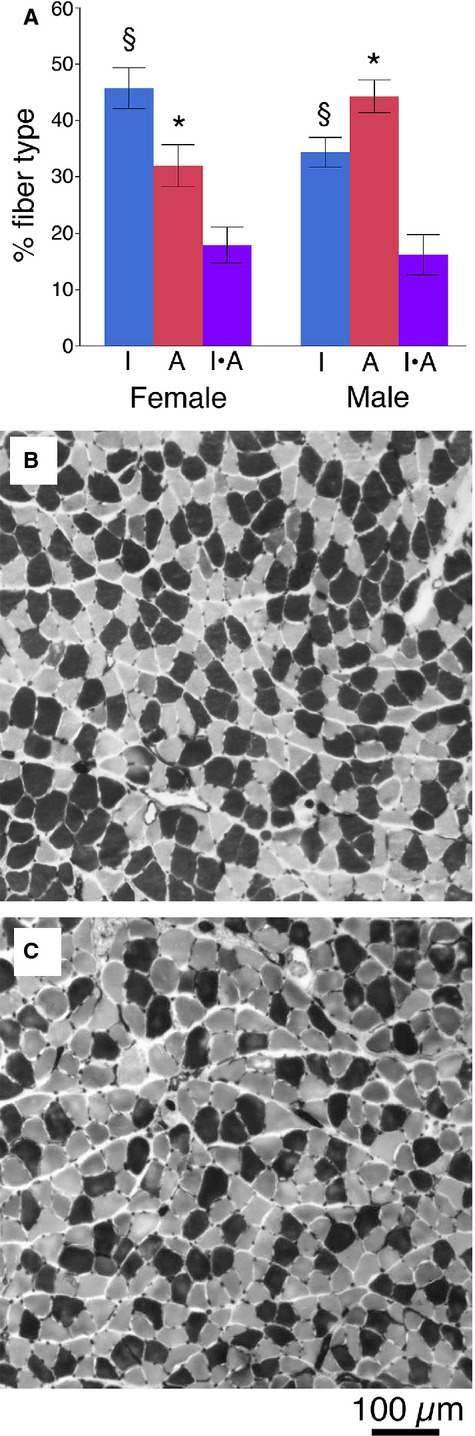
Differences in proportions of type I fibers in male and female soleus muscles. (A) Proportions of type IIA fibers are significantly greater in male soleus (*P < 0.015), whereas the proportion of type I fibers is significantly greater in the female soleus (^§^*P* < 0.010). No differences were detected between the proportions of I/IIA hybrids (*P* > 0.70). Histochemical staining confirms that female male mice (B) typically have a higher proportion of type I fibers (dark) in the soleus compared with males (C).

The sex‐specific difference detected by the ANOVA was also observed with *t*‐tests comparing the sexes independent of diet (Fig. 6A). Female mice had a greater number of type I fibers in the soleus compared with males (female: 45.8 ± 3.6% vs. male: 34.4 ± 2.6%; *P* < 0.01), while male mice possessed a significantly greater proportion of IIA fibers (female: 32.0 ± 3.7% vs. male: 44.3 ± 2.9%; *P* < 0.009). This pattern is consistent with complementary histochemical analyses in our laboratory (Fig. 6B and C).

**Figure 7.**
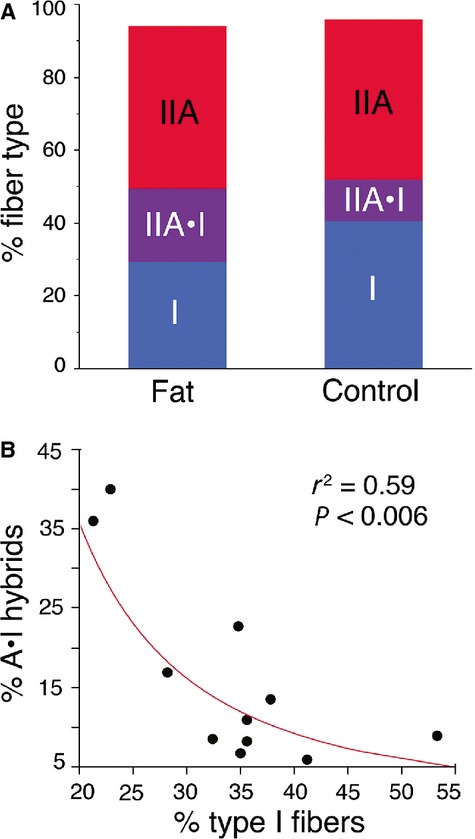
Decline in type I fibers is coincident with an increase in type I/IIA hybrids in male soleus. (A) The relative proportions of type I and I/IIA hybrids combined are not different between the high‐fat (Fat) and control diet mice (Control) (*t*‐test, *P* > 0.31). In both groups, >90% of the fibers are I, I/IIA hybrid, or IIA. (B) The relative proportions of type I and I/IIA hybrid fibers are inversely correlated with one another (*r*^2^ = 0.059, *P* < 0.006).

In the male mice, >90% of the soleus fibers were either type I, I/IIA, or IIA. The collective total of these fibers types did not differ between fat and control mice (Fat: 94.1 ± 1.1% vs. Control: 95.8 ± 2.3%; *P* > 0.51) (Fig. 7A). Although the I/IIA hybrid proportions were not statistically different from one another in these groups (Fat: 20.1 ± 5.8% vs. Control: 11.5 ± 6.9%; *P* < 0.12), the proportions of type I fibers and type I/IIA hybrids were inversely correlated (I/IIA% = 2.22 + (%I)^−1.96^; *r*^2^ = 0.59; *P* < 0.006) (Fig. 7B).

### Proportions of type I and IIA MHC in male hybrids

**Figure 8.**
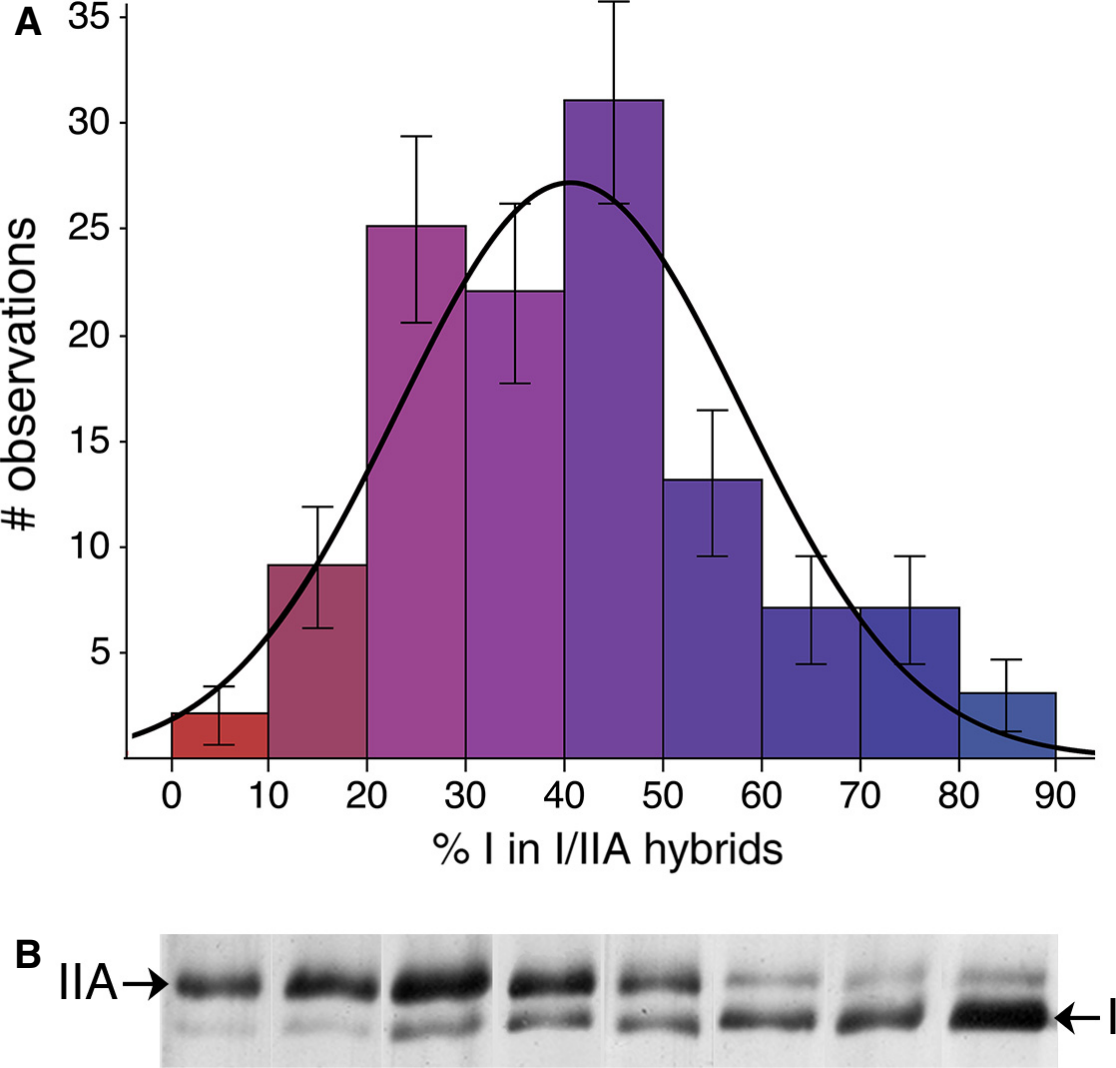
Relative proportions of types I and IIA MHC isoforms within I/IIA hybrids from the male soleus. (A) The proportions of isoforms conformed to a pattern similar to a normal distribution, meaning that the majority of hybrids contained roughly equal proportions of the two MHC isoforms (the curve is a normal distribution fit to the data). (B) Examples of SDS‐PAGE identification of I/IIA hybrids arranged as a continuum from hybrids with large proportions of type IIA MHC (left), to those containing mostly type I (right).

Given that the loss of type I fibers in male mice appeared to correspond with an increase in the I/IIA hybrid fiber population, we were interested in determining whether the composition of these hybrids might differ between fat and control mice. Specifically, we anticipated that if type I fibers had been converted into I/IIA hybrids, these hybrids might possess a higher composition of the type I MHC than in the control fibers. Our results did not confirm this hypothesis, as the hybrids from both groups contained an average of ~40% type I MHC (Fat: 40.6 + 1.9% vs. Control: 40.3 + 2.3%; *P* > 0.92). Overall, the relative proportions of MHC isoforms were very close to being normally distributed (Fig. 8). There was no correlation between the proportion of type I MHC in these hybrids and any measure of body fat.

## Discussion

We observed important interactions between sex and adiposity reflected in the fiber types within the soleus muscles (Table 1; Fig. 5). Specifically, male mice fed a high‐fat diet possessed significantly fewer type I fibers than control males (Fig. 5A). Moreover, the relative proportions of these type I fibers in male mice were inversely correlated with the amount of body fat (Fig. 5B). Our data suggest that these type I fibers were principally replaced in number by the I/IIA hybrids, as the proportions of these hybrids were inversely correlated with type I fiber proportions (Fig. 7). This makes sense in the context of Pette's (2006) nearest‐neighbor hypothesis, which poses that as fibers shift from one type to another, they sequentially transition through hybrids toward the next fastest fiber type. Our interpretation is that the high‐fat diet in males resulted in a transformation of some type I fibers into I/IIA hybrids, with smaller numbers of these hybrids possibly being transformed into pure IIA fibers: I **→** I/IIA **→** IIA. Previous authors have discussed the possibility that changes in activity, rather than obesity per se, might be responsible for shifts in muscle phenotype in genetically obese mice (Stickland et al. 1994; Tankersly et al. 1998; Warmington et al. 2000; Kemp et al. 2009). Although we did not collect behavioral data in this study, we do not believe that activity levels were responsible for the observed fiber type shifts. In another recent study, we exercised C57 BLK/6J mice on a treadmill for 6 weeks, which resulted in a significant increase in muscle mass but no change in fiber type (Glaser et al. 2010). As there were no differences in muscle mass in the current study (Fig. 2C), it is unlikely that the altered fiber types in the soleus resulted from reduced activity levels.

In the current study, we used single fiber SDS‐PAGE to precisely identify several major fiber types in the soleus and plantaris muscles of fat and control mice. These two shank muscles provided a comprehensive sample of the fiber types commonly found in mouse muscles, as the soleus is predominantly formed of slower fiber types (I, I/IIA, and IIA fibers), whereas the plantaris is comprised mostly of faster fiber types (IIX, IIX/IIB, and IIB fibers) (Fig. 4). Single fiber SDS‐PAGE has been advocated as the “gold‐standard” for fiber type identification because it provides the most objective and quantitative method for identifying fiber types (Pandorf et al. 2010). In this respect, our study is fairly unique because previous studies focusing on fiber‐type changes with obesity have almost exclusively depended on ATP histochemistry to identify fiber types (Kemp et al. 2009 is an exception). These techniques can broadly differentiate among fiber types, but are incapable of accurately identifying hybrid fibers. In the current study, we would not have been able to detect the reported changes in the soleus muscles using histochemistry alone. Another aspect of this study that sets it apart from previous works is that we maintained mice on a high‐fat diet for approximately 1 year, whereas previous studies on diet‐induced obesity in C57 BLK/6J mice have lasted in the range of a few days to 20 weeks (Turner et al. 2007; de Wilde et al. 2008, 2009; Shortreed et al. 2009). It may be that shorter studies have failed to detect fiber‐type changes because fiber‐type transitions are relatively slow processes that require significant fiber remodeling.

We also found that sex in itself had a significant impact on the fiber‐type composition of the soleus muscles, with female mice having significantly more type I fibers than males (Table 1; Fig. 6). In fact, the relative proportions of the type I and IIA fibers were essentially reversed with respect to one another in the males and females, with no discernable differences in the proportions of I/IIA hybrids (Fig. 6A). This pattern is consistent with many other studies reporting sex‐specific differences in the muscles of mice (Eason et al. 2000; Hartmann et al. 2001), rats (Drzymala‐Celichowska et al. 2012), rabbits (English et al. 1999; Reader et al. 2001; English and Schwartz 2002; English and Widmer 2003), and humans (Simoneau et al. 1985; Simoneau and Bouchard 1989; Staron et al. 2000; Maher et al. 2009). The shift toward type I fibers in females compared with males is a general theme of each of these studies, and several studies have implicated testosterone in these sex‐specific differences (Holmäng et al. 1990; Mårin et al. 1994; Reader et al. 2001). In the current study, the response of type I fibers to the high‐fat diet shows that these fibers may retain a level of physiological responsiveness that sets them apart from the same fibers in females. Previous studies of fiber‐type changes with obesity in mice have focused exclusively on males (Almond and Enser 1984; Stickland et al. 1994; Warmington et al. 2000; Kemp et al. 2009; Sainz et al. 2009; Shortreed et al. 2009), or failed to report the sex of the animals (Tankersly et al. 1998). The significant interactions between sex and obesity in affecting fiber type represent an important pattern that should guide future research.

The significant reductions in type I fibers (Fig. 5) are consistent with patterns that have been well documented in studies of human skeletal muscles over the past 30 years. Early studies reported significant correlations among skeletal muscle composition, adiposity, and metabolic substrate utilization (Lithell et al. 1981; Lillioja et al. 1987; Wade et al. 1990). Subsequent studies of muscle fiber type in patients undergoing gastric bypass surgery confirmed the inverse correlation between type I fiber proportions and body fat (Hickey et al. 1995; Tanner et al. 2002). Collectively, an extensive body of studies have documented the significant inverse correlation between type I fiber proportions and the amount of body fat (Mårin et al. 1994; Kriketos et al. 1996, 1997; Helge et al. 1999; Gaster et al. 2001; Oberbach et al. 2006; Stuart et al. 2013). Many these studies also demonstrated functional linkages between fiber composition including glucose uptake and maximum oxygen consumption, with obesity leading to significant impairments in these processes (Mårin et al. 1994; Kriketos et al. 1996, 1997; Nyholm et al. 1997; Helge et al. 1999; Gaster et al. 2001; Stuart et al. 2013). Although our study design limits our ability to infer the functionality of changes within the muscles, our data are consistent with the correlations between type I fiber proportions and obesity. An important difference between our results and those from human studies is that we failed to detect any changes in the proportions of fast fiber types in the mouse, whereas human studies have reported significant increases in fast glycolytic fibers as a result of obesity (Mårin et al. 1994; Kriketos et al. 1997; Oberbach et al. 2006).

Results from rodent studies of muscle fiber types in obesity have been more variable than those from human studies, in part because of differences in experimental design and the animal models used. Shortreed et al. (2009) detected significant impairments in glucose and fatty acid in single isolated muscle fibers of high‐fat diet–induced obese C57 BLK/6J mice, as well as impaired insulin‐stimulated glycogen synthesis. However, they reported only a minor shift in fiber type toward slower fiber types (IIB → IIA fibers in the gastrocnemius/plantaris). In a more recent study from this group, they observed an increase in the oxidative capacity of skeletal muscles after 3 weeks and a similar shift in fiber types (Trajcevski et al. 2013). They proposed that this early increase in oxidative capacity essentially “fails” as mice age and develop insulin resistance (Trajcevski et al. 2013). Both the Shortreed et al. (2009) study and another by Turpin et al. (2009) reported increases in the soleus fiber diameters of high‐fat diet–fed mice, but with no changes in fiber‐type proportions. In a similar study, Turner et al. (2007) reported that muscles of high‐fat diet–fed C57 BLK/6J mice exhibited an up‐regulation of PGC‐1α and other indicators of enhanced oxidative capacity of the fibers, but fiber types were not reported. de Wilde et al. (2008) focused on short‐term changes in mouse skeletal muscle transcripts and proteins in response to a high‐fat diet containing palm oil. They reported a significant shift toward slower fiber type genes and the machinery of oxidative metabolism in muscles of the quadriceps complex, but in a follow‐up study they detected only minor changes in the muscle transcriptomes of mice fed a high‐fat diet for 8 weeks (de Wilde et al. 2009). Collectively, these studies of diet‐induced obesity in C57 BLK/6J mice suggest that changes in muscle fiber type are minor, but that a high‐fat diet leads to changes in oxidative capacity of the muscles. The differences between the findings of these results and ours may stem from the relatively shorter time periods of these studies (3 days–20 weeks), whereas ours is based on mice maintained on a high‐fat diet for a full year.

Several studies of have also focused on the skeletal muscles of the *ob/ob* leptin knockout mouse (Almond and Enser 1984; Stickland et al. 1994; Tankersly et al. 1998; Warmington et al. 2000; Kemp et al. 2009; Sainz et al. 2009). The most consistent observation in these mice is that their muscle size is significantly reduced (Almond and Enser 1984; Stickland et al. 1994; Warmington et al. 2000; Kemp et al. 2009; Sainz et al. 2009), specifically within the fast type IIB and IIX fiber populations (Almond and Enser 1984; Tankersly et al. 1998; Warmington et al. 2000; Kemp et al. 2009). Some studies have also reported a shift in fiber‐type proportions away from the IIB fibers and toward slower fiber types, but fiber‐type determinations from whole muscle homogenates cannot pinpoint whether this difference is due to fiber‐type conversion, or from the already noted atrophy of the faster fiber types (Tankersly et al. 1998; Kemp et al. 2009). Kemp et al. (2009) reported a higher proportion of hybrid fibers in the *ob/ob* mice than in controls, and proposed that these hybrids represented a shift toward slower, more aerobic fiber types. Collectively, the patterns from these *ob/ob* mice are in stark contrast to our findings of no differences in muscle mass, but with a shift toward faster fiber types in C57 BLK/6J mice. The juxtaposition between our results and those from the leptin knockout mice suggests that obesity per se might not be the cause changes in muscle fiber type, but these alterations may be linked more directly to the effects of leptin itself. In support of this possibility, leptin treatment reverses the effects seen in the *ob/ob* mice, causing a shift toward faster fiber types and an increase in muscle mass (Tankersly et al. 1998; Warmington et al. 2000; Sainz et al. 2009). Further studies are needed to differentiate between the effects of obesity, leptin, and other physiological parameters in these mice.

The extensive single fiber sampling techniques used in the current study limit the number of different muscles that can be effectively sampled. However, this detailed approach provides a degree of fiber‐type resolution not possible from histochemical techniques alone, and we were able to assess a complete range of fiber types from the mouse hindlimb. In the soleus of obese male mice, type I fibers appear to have been converted into the faster type I/IIA hybrid fibers. Moreover, the relative proportions of type I fibers were inversely correlated with body fat levels. These results are highly consistent with patterns observed in human studies, where obesity is associated with significantly lower proportions of type I fibers in favor of a higher number of type II fibers. Further studies are needed to elucidate the mechanisms responsible for these differences in muscle phenotype.

## Acknowledgments

Many thanks to Kathryn Medler at the University at Buffalo for generously providing the muscles used in these analyses.

## Conflict of Interest

None declared.
